# [1,3-Bis(diphenyl­phosphino)propane-κ^2^ *P*,*P*′]diiodido(perfluoro­propyl)rhodium(III) dichloro­methane solvate

**DOI:** 10.1107/S1600536808029978

**Published:** 2008-09-27

**Authors:** Basu Panthi, Stephen L. Gipson, Andreas Franken

**Affiliations:** a Chemistry Department, Baylor University, One Bear Place 97348, Waco, TX 76798, USA

## Abstract

The structure of the title compound, [RhI_2_(C_3_F_7_)(C_27_H_26_P_2_)]·CH_2_Cl_2_, at 110 (2) K is an unusual example of a structurally characterized square-based pyramidal alkyl complex of rhodium(III). The Rh—C bond is relatively short at 1.996 (6) Å. This short metal–carbon bond length is typical of perfluoro complexes of transition metals and illustrates the enhanced bond strength in these compounds.

## Related literature

The most closely related structure is that of *trans*-Rh(CF_2_H)(PPh_3_)_2_Cl_2_ (Burrell *et al.*, 1990 ▶). For similar square-based pyramidal Rh^III^ structures, see: Søtofte & Hjortkjær (1994 ▶); McGuiggan *et al.* (1980 ▶); Egglestone *et al.* (1977 ▶); Shie *et al.* (1989 ▶); Moloy & Petersen (1995 ▶). For perfluoro­alkyl Rh^III^ complexes having pseudo-octa­hedral piano-stool geometries, see: Churchill (1965 ▶); Hughes, Kovacik *et al.* (2001 ▶); Hughes *et al.* (1997 ▶); Bowden *et al.* (2002 ▶); Hughes, Lindner *et al.* (2001 ▶). For more information on bonding in perfluoro­alkyl transition metal complexes, see: Gunawardhana *et al.* (2008 ▶).
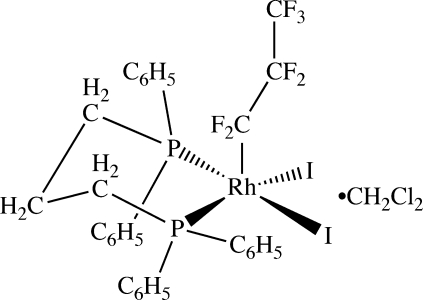


## Experimental

### 

#### Crystal data

[RhI_2_(C_3_F_7_)(C_27_H_26_P_2_)]·CH_2_Cl_2_
*M* *_r_* = 1023.08
Monoclinic, 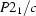

*a* = 14.0419 (6) Å
*b* = 15.1273 (6) Å
*c* = 17.7722 (7) Å
β = 110.299 (2)°
*V* = 3540.6 (2) Å^3^
*Z* = 4
Mo *K*α radiation
μ = 2.53 mm^−1^
*T* = 110 (2) K
0.19 × 0.09 × 0.08 mm

#### Data collection

Bruker Nonius X8 APEX CCD area-detector diffractometer
Absorption correction: multi-scan (*SADABS*; Bruker, 2003 ▶) *T*_min_ = 0.644, *T*_max_ = 0.833
56038 measured reflections
6467 independent reflections
5127 reflections with *I* > 2σ(*I*)
*R*_int_ = 0.076

#### Refinement

*R*[*F* ^2^ > 2σ(*F* ^2^)] = 0.041
*wR*(*F* ^2^) = 0.089
*S* = 1.04
6467 reflections
406 parameters
H-atom parameters constrained
Δρ_max_ = 1.72 e Å^−3^
Δρ_min_ = −0.89 e Å^−3^

### 

Data collection: *APEX2* (Bruker, 2003 ▶); cell refinement: *APEX2*; data reduction: *APEX2*; program(s) used to solve structure: *SHELXTL* (Sheldrick, 2008 ▶); program(s) used to refine structure: *SHELXTL*; molecular graphics: *SHELXTL*; software used to prepare material for publication: *SHELXTL*.

## Supplementary Material

Crystal structure: contains datablocks I, global. DOI: 10.1107/S1600536808029978/kj2099sup1.cif

Structure factors: contains datablocks I. DOI: 10.1107/S1600536808029978/kj2099Isup2.hkl

Additional supplementary materials:  crystallographic information; 3D view; checkCIF report

## Figures and Tables

**Table:** 

I1—Rh1	2.6920 (6)
I2—Rh1	2.6743 (6)
Rh1—C4	1.996 (6)
Rh1—P2	2.3185 (15)
Rh1—P1	2.3248 (15)

**Table:** 

C4—Rh1—P2	89.82 (18)
C4—Rh1—P1	96.10 (19)
P2—Rh1—P1	92.02 (5)
C4—Rh1—I2	99.44 (18)
P2—Rh1—I2	88.04 (4)
P1—Rh1—I2	164.46 (4)
C4—Rh1—I1	103.92 (17)
P2—Rh1—I1	166.17 (4)
P1—Rh1—I1	88.17 (4)
I2—Rh1—I1	88.105 (17)

**Fig. 1.**
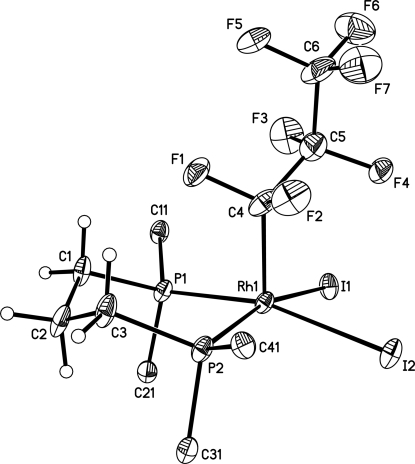
A view of the title compound with the atom-numbering scheme. Displacement ellipsoids are drawn at the 40% probability level. The phenyl rings and the solvent CH2Cl2 have been omitted for clarity.

## supplementary crystallographic information

### Comment

The stucture of the title compound is shown in Fig. 1. The geometry about the rhodium atom is square-based pyramidal (sbp) with the perfluoropropyl group occupying the axial position. This geometry is similar to that of *trans*-Rh(CF_2_H)(PPh_3_)_2_Cl_2_ (Burrell *et al.*, 1990), *cis*-Rh(COMe)(dppp)I_2_, where dppp = 1,3-bis(diphenylphosphino)propane (Søtofte & Hjortkjaer, 1994), *cis*-Rh(COPh)(dppp)Cl_2_ (McGuiggan *et al.*, 1980), *trans*-Rh(COCH_2_CH_2_Ph)(PPh_3_)_2_Cl_2_ (Egglestone *et al.*, 1977), and *cis*-Rh(COCH_2_CH_3_)(PPh_3_)_2_Cl_2_ (Shie *et al.*, 1989), without the significant distortion toward trigonal bipyramidal geometry reported for *cis*-Rh(COCH_3_)(dppp)I_2_ by Moloy & Petersen (1995). The structure of the title compound includes one CH_2_Cl_2_ solvent molecule, not shown in Fig. 1. We wish to report here the structure of this unique species, though complete characterization is not possible at this time due to our inability to find suitable methods for its reliable isolation and purification.

So far as we can determine, the title compound is only the second structure of a sbp alkyl–Rh(III) complex of the class Rh(*R*)(phosphine)_2_*X*_2_ (*X* = halide). Numerous structures have been reported for sbp acyl complexes of this type, and there are quite a few published examples of alkyl–Rh(III) complexes with pseudo-octahedral piano stool geometries, including several perfluoroalkyl complexes of the type CpRh(*R*_f_)(*L*)*X* (Cp = cyclopentadienyl, pentamethylcyclopentadienyl, tris(pyrazolyl)borate; *R*_f_ = perfluoroethyl, perfluoropropyl; *L* = CO, PMe_3_; *X* = Cl, H, H_2_O) (Churchill, 1965; Hughes, Kovacik *et al.*, 2001; Hughes *et al.*, 1997; Bowden *et al.*, 2002; Hughes, Lindner *et al.*, 2001). The importance of the title compound is that it sheds additional light on the bonding in perfluoroalkyl transition metal complexes. The Rh—C bond length of the title compound (1.996 (6) Å) compares favorably with that of the difluoromethyl complex (1.98 Å) and the sbp Rh(III)–acyl complexes (1.95–2.0 Å), but is somewhat shorter than those in the perfluoropropyl piano stool complexes (2.05–2.09 Å). While there has been some discussion as to whether the bond shortening observed for perfluoroalkyl and acyl ligands can be attributed to metal to ligand back-bonding (Moloy & Petersen, 1995), this is clearly not the case in comparing perfluoropropyl–Rh(III) complexes with sbp and piano stool geometries. Unfortunately, there are no reported structures for hydrocarbon Rh(III)–alkyl complexes with which to compare the title compound. The shortening of the metal–carbon bonds in perfluoroalkyl transition metal complexes, and the concomitant strengthening of this bond, has previously been explained in terms of electrostatic effects caused by the relatively large positive charge on the α-carbon of the perfluoroalkyl group (Gunawardhana *et al.*, 2008).

### Experimental

Chlorodicarbonylrhodium(I) dimer, [Rh(CO)_2_Cl]_2_, (Strem Chemicals, 0.259 g, 1.34 mmol) was taken in a 100 ml round bottom flask into a nitrogen-atmosphere glove box and 12.5 ml of acetone was added. Then a solution of 0.216 g (1.44 mmol) of NaI in 7.5 ml of acetone was added and the mixture was stirred for about one hour. After that a solution of 1,3-bis(diphenylphosphino)propane (Strem Chemicals, 0.590 g, 1.43 mmol) in 7.5 ml of acetone was added. After about 3 h the round bottom flask was taken out of the glove box and the solution was concentrated under reduced pressure, forming a yellow precipitate of Rh(CO)(dppp)I. This product was collected by filtration, washed with methanol and dried overnight in a vacuum oven at room temperature. A portion of this Rh(CO)(dppp)I (0.236 g, 0.35 mmol), NaI (0.358 g, 2.39 mmol) and heptafluorobutyryl chloride, C_3_F_7_COCl, (Acros Organics, 0.137 g, 0.56 mmol) were added to 10 ml of methylene chloride in a 100 ml Schlenk flask and their reaction was monitored by IR. Initially peaks were observed at 2056, 1996, 1789 and 1695 cm^-1^. The solution was stirred until only an IR absorption at 2075 cm^-1^ remained. The solution was then filtered, the filtrate was concentrated under reduced pressure and a precipitate was obtained by the addition of hexane. NMR spectroscopy showed the precipitate to be impure and attempts at purification by chromatography failed. Finally, a small amount of the impure product was dissolved in methylene chloride, layered with hexanes and stored in a freezer for about four months. Single crystals of the title compound resulted from this treatment.

### Refinement

All of the hydrogen atoms were set riding on their parent carbon atoms in calculated positions and were assigned fixed isotropic thermal parameters calculated as *U*_iso_(H) = 1.2*U*_iso_(C). Phenyl-H atoms were set riding with C—H = 0.95 Å and dppp bridge H atoms with C—H = 0.99 Å. The residual density extrema result from a very slight disorder in the C_3_F_7_ ligand and are located in its vicinity.

### Crystal data

**Table:**

[RhI_2_(C_3_F_7_)(C_27_H_26_P_2_)]·CH_2_Cl_2_	*F*(000) = 1968
*M**_r_* = 1023.08	*D*_x_ = 1.919 Mg m^−^^3^
Monoclinic, *P*2_1_/*c*	Mo *K*α radiation, λ = 0.71073 Å
Hall symbol: -P 2ybc	Cell parameters from 7865 reflections
*a* = 14.0419 (6) Å	θ = 2.4–23.9°
*b* = 15.1273 (6) Å	µ = 2.53 mm^−^^1^
*c* = 17.7722 (7) Å	*T* = 110 K
β = 110.299 (2)°	Needle, orange
*V* = 3540.6 (2) Å^3^	0.19 × 0.09 × 0.08 mm
*Z* = 4	

### Data collection

**Table:**

Bruker Nonius X8 APEX CCD area-detector diffractometer	6467 independent reflections
Radiation source: fine-focus sealed tube	5127 reflections with *I* > 2σ(*I*)
graphite	*R*_int_ = 0.077
φ and ω scans	θ_max_ = 25.4°, θ_min_ = 1.8°
Absorption correction: multi-scan (SADABS; Bruker, 2003)	*h* = −16→16
*T*_min_ = 0.644, *T*_max_ = 0.833	*k* = −18→18
56038 measured reflections	*l* = −21→21

### Refinement

**Table:**

Refinement on *F*^2^	Primary atom site location: structure-invariant direct methods
Least-squares matrix: full	Secondary atom site location: difference Fourier map
*R*[*F*^2^ > 2σ(*F*^2^)] = 0.041	Hydrogen site location: inferred from neighbouring sites
*wR*(*F*^2^) = 0.089	H-atom parameters constrained
*S* = 1.04	*w* = 1/[σ^2^(*F*_o_^2^) + (0.0249*P*)^2^ + 19.6317*P*] where *P* = (*F*_o_^2^ + 2*F*_c_^2^)/3
6467 reflections	(Δ/σ)_max_ = 0.001
406 parameters	Δρ_max_ = 1.72 e Å^−^^3^
0 restraints	Δρ_min_ = −0.89 e Å^−^^3^

### Special details

**Table:**

Geometry. All e.s.d.'s (except the e.s.d. in the dihedral angle between two l.s. planes) are estimated using the full covariance matrix. The cell e.s.d.'s are taken into account individually in the estimation of e.s.d.'s in distances, angles and torsion angles; correlations between e.s.d.'s in cell parameters are only used when they are defined by crystal symmetry. An approximate (isotropic) treatment of cell e.s.d.'s is used for estimating e.s.d.'s involving l.s. planes.
Refinement. Refinement of *F*^2^ against ALL reflections. The weighted *R*-factor *wR* and goodness of fit *S* are based on *F*^2^, conventional *R*-factors *R* are based on *F*, with *F* set to zero for negative *F*^2^. The threshold expression of *F*^2^ > σ(*F*^2^) is used only for calculating *R*-factors(gt) *etc*. and is not relevant to the choice of reflections for refinement. *R*-factors based on *F*^2^ are statistically about twice as large as those based on *F*, and *R*- factors based on ALL data will be even larger.

### Fractional atomic coordinates and isotropic or equivalent isotropic displacement parameters (Å^2^)

**Table:**

	*x*	*y*	*z*	*U*_iso_*/*U*_eq_	
I1	0.40578 (3)	0.70275 (3)	0.16010 (2)	0.02613 (11)	
I2	0.17802 (3)	0.84549 (3)	0.06878 (2)	0.02804 (11)	
Rh1	0.26222 (3)	0.76934 (3)	0.21248 (3)	0.01917 (11)	
Cl1	−0.21560 (15)	0.72551 (15)	−0.03714 (13)	0.0578 (5)	
Cl2	−0.09542 (19)	0.81404 (16)	−0.12019 (13)	0.0693 (6)	
P1	0.36657 (11)	0.73432 (10)	0.34229 (9)	0.0210 (3)	
P2	0.15974 (11)	0.85820 (10)	0.25875 (9)	0.0222 (3)	
F1	0.1605 (3)	0.6371 (3)	0.2698 (2)	0.0382 (9)	
F2	0.0631 (3)	0.6945 (3)	0.1569 (3)	0.0465 (10)	
F3	0.2602 (3)	0.5426 (3)	0.1897 (3)	0.0515 (11)	
F4	0.1739 (3)	0.6128 (3)	0.0763 (2)	0.0405 (10)	
F5	0.0728 (3)	0.4927 (3)	0.1982 (3)	0.0567 (12)	
F6	0.1130 (3)	0.4474 (3)	0.0965 (3)	0.0611 (12)	
F7	−0.0005 (3)	0.5454 (3)	0.0795 (3)	0.0596 (12)	
C1	0.3056 (5)	0.7314 (4)	0.4179 (3)	0.0273 (14)	
H1A	0.2595	0.6797	0.4069	0.033*	
H1B	0.3587	0.7220	0.4710	0.033*	
C2	0.2453 (5)	0.8132 (4)	0.4224 (3)	0.0310 (15)	
H2A	0.2308	0.8116	0.4731	0.037*	
H2B	0.2874	0.8662	0.4239	0.037*	
C3	0.1458 (5)	0.8222 (4)	0.3528 (3)	0.0303 (15)	
H3A	0.1022	0.8652	0.3677	0.036*	
H3B	0.1105	0.7644	0.3438	0.036*	
C4	0.1655 (5)	0.6691 (4)	0.2002 (4)	0.0308 (15)	
C5	0.1734 (5)	0.5873 (5)	0.1490 (4)	0.0410 (17)	
C6	0.0904 (6)	0.5178 (5)	0.1333 (5)	0.0447 (19)	
C11	0.4393 (4)	0.6312 (4)	0.3639 (3)	0.0240 (13)	
C12	0.5436 (4)	0.6318 (4)	0.3805 (3)	0.0256 (13)	
H12	0.5774	0.6856	0.3784	0.031*	
C13	0.5984 (5)	0.5530 (4)	0.4002 (4)	0.0303 (15)	
H13	0.6700	0.5537	0.4133	0.036*	
C14	0.5499 (5)	0.4747 (4)	0.4007 (4)	0.0332 (15)	
H14	0.5877	0.4213	0.4130	0.040*	
C15	0.4454 (5)	0.4732 (4)	0.3834 (4)	0.0378 (16)	
H15	0.4117	0.4189	0.3841	0.045*	
C16	0.3908 (5)	0.5514 (4)	0.3652 (4)	0.0313 (15)	
H16	0.3195	0.5505	0.3534	0.038*	
C21	0.4605 (4)	0.8225 (4)	0.3763 (3)	0.0226 (13)	
C22	0.4951 (5)	0.8681 (4)	0.3224 (4)	0.0295 (14)	
H22	0.4700	0.8521	0.2673	0.035*	
C23	0.5641 (5)	0.9351 (4)	0.3471 (4)	0.0370 (16)	
H23	0.5869	0.9648	0.3094	0.044*	
C24	0.6008 (5)	0.9598 (4)	0.4270 (4)	0.0371 (16)	
H24	0.6484	1.0067	0.4441	0.044*	
C25	0.5686 (5)	0.9164 (5)	0.4810 (4)	0.0389 (17)	
H25	0.5942	0.9329	0.5360	0.047*	
C26	0.4990 (5)	0.8486 (4)	0.4562 (3)	0.0324 (15)	
H26	0.4769	0.8192	0.4945	0.039*	
C31	0.2158 (4)	0.9679 (4)	0.2814 (3)	0.0240 (13)	
C32	0.2921 (5)	0.9957 (4)	0.2548 (4)	0.0289 (14)	
H32	0.3173	0.9563	0.2246	0.035*	
C33	0.3326 (5)	1.0796 (5)	0.2713 (4)	0.0364 (16)	
H33	0.3856	1.0973	0.2528	0.044*	
C34	0.2961 (5)	1.1377 (4)	0.3145 (4)	0.0345 (15)	
H34	0.3243	1.1953	0.3265	0.041*	
C35	0.2180 (5)	1.1117 (5)	0.3404 (4)	0.0396 (17)	
H35	0.1912	1.1520	0.3688	0.048*	
C36	0.1792 (5)	1.0274 (4)	0.3249 (4)	0.0335 (15)	
H36	0.1269	1.0095	0.3440	0.040*	
C41	0.0291 (4)	0.8820 (4)	0.1963 (3)	0.0226 (13)	
C42	−0.0519 (5)	0.8303 (4)	0.1997 (4)	0.0287 (14)	
H42	−0.0388	0.7795	0.2333	0.034*	
C43	−0.1512 (5)	0.8523 (5)	0.1544 (4)	0.0389 (17)	
H43	−0.2057	0.8170	0.1574	0.047*	
C44	−0.1708 (5)	0.9251 (5)	0.1052 (4)	0.0395 (17)	
H44	−0.2387	0.9398	0.0736	0.047*	
C45	−0.0917 (5)	0.9766 (5)	0.1018 (4)	0.0372 (16)	
H45	−0.1057	1.0271	0.0680	0.045*	
C46	0.0080 (5)	0.9563 (4)	0.1470 (4)	0.0282 (14)	
H46	0.0617	0.9928	0.1443	0.034*	
C51	−0.0967 (5)	0.7705 (5)	−0.0287 (4)	0.0471 (19)	
H51A	−0.0442	0.7239	−0.0102	0.057*	
H51B	−0.0793	0.8179	0.0122	0.057*	

### Atomic displacement parameters (Å^2^)

**Table:**

	*U*^11^	*U*^22^	*U*^33^	*U*^12^	*U*^13^	*U*^23^
I1	0.0243 (2)	0.0355 (2)	0.0208 (2)	0.00564 (17)	0.01062 (16)	−0.00196 (16)
I2	0.0282 (2)	0.0369 (2)	0.0225 (2)	0.00667 (18)	0.01317 (17)	0.01052 (17)
Rh1	0.0193 (2)	0.0227 (2)	0.0189 (2)	0.00168 (19)	0.01094 (19)	0.00419 (18)
Cl1	0.0378 (11)	0.0687 (14)	0.0676 (14)	−0.0103 (10)	0.0192 (10)	−0.0100 (11)
Cl2	0.0813 (16)	0.0722 (15)	0.0477 (12)	−0.0137 (13)	0.0138 (12)	0.0155 (11)
P1	0.0232 (8)	0.0236 (8)	0.0193 (8)	0.0041 (6)	0.0113 (6)	0.0057 (6)
P2	0.0225 (8)	0.0263 (8)	0.0219 (8)	0.0065 (6)	0.0130 (7)	0.0068 (6)
F1	0.036 (2)	0.049 (2)	0.033 (2)	−0.0120 (18)	0.0163 (17)	0.0093 (17)
F2	0.037 (2)	0.043 (2)	0.066 (3)	0.0145 (19)	0.027 (2)	0.009 (2)
F3	0.044 (3)	0.052 (3)	0.057 (3)	0.025 (2)	0.015 (2)	0.002 (2)
F4	0.047 (2)	0.047 (2)	0.029 (2)	−0.0219 (19)	0.0155 (18)	0.0021 (17)
F5	0.073 (3)	0.051 (3)	0.055 (3)	−0.029 (2)	0.033 (2)	0.005 (2)
F6	0.067 (3)	0.044 (3)	0.079 (3)	−0.004 (2)	0.034 (3)	−0.018 (2)
F7	0.039 (3)	0.066 (3)	0.075 (3)	−0.003 (2)	0.020 (2)	0.002 (2)
C1	0.029 (3)	0.039 (4)	0.018 (3)	0.011 (3)	0.014 (3)	0.008 (3)
C2	0.039 (4)	0.039 (4)	0.024 (3)	0.017 (3)	0.021 (3)	0.016 (3)
C3	0.034 (4)	0.040 (4)	0.025 (3)	0.017 (3)	0.021 (3)	0.011 (3)
C4	0.027 (3)	0.030 (4)	0.043 (4)	0.007 (3)	0.022 (3)	0.015 (3)
C5	0.045 (4)	0.045 (4)	0.037 (4)	−0.007 (4)	0.019 (3)	0.003 (3)
C6	0.052 (5)	0.037 (4)	0.060 (5)	−0.005 (4)	0.038 (4)	0.010 (4)
C11	0.026 (3)	0.027 (3)	0.022 (3)	0.006 (3)	0.012 (3)	0.003 (2)
C12	0.026 (3)	0.031 (3)	0.022 (3)	0.005 (3)	0.012 (3)	0.003 (3)
C13	0.029 (4)	0.037 (4)	0.025 (3)	0.009 (3)	0.011 (3)	0.008 (3)
C14	0.045 (4)	0.031 (4)	0.029 (4)	0.012 (3)	0.020 (3)	0.006 (3)
C15	0.048 (4)	0.025 (4)	0.043 (4)	0.004 (3)	0.019 (3)	0.010 (3)
C16	0.030 (4)	0.035 (4)	0.034 (4)	0.003 (3)	0.016 (3)	0.008 (3)
C21	0.025 (3)	0.022 (3)	0.021 (3)	0.009 (2)	0.009 (3)	0.002 (2)
C22	0.029 (3)	0.037 (4)	0.019 (3)	−0.006 (3)	0.004 (3)	−0.004 (3)
C23	0.040 (4)	0.036 (4)	0.036 (4)	−0.009 (3)	0.015 (3)	0.002 (3)
C24	0.030 (4)	0.030 (4)	0.044 (4)	−0.003 (3)	0.004 (3)	−0.009 (3)
C25	0.043 (4)	0.038 (4)	0.025 (4)	0.008 (3)	−0.002 (3)	−0.007 (3)
C26	0.043 (4)	0.035 (4)	0.018 (3)	0.006 (3)	0.009 (3)	0.003 (3)
C31	0.026 (3)	0.028 (3)	0.020 (3)	0.005 (3)	0.011 (3)	0.005 (2)
C32	0.034 (4)	0.028 (3)	0.027 (3)	−0.001 (3)	0.014 (3)	−0.002 (3)
C33	0.041 (4)	0.041 (4)	0.033 (4)	−0.005 (3)	0.018 (3)	−0.001 (3)
C34	0.032 (4)	0.034 (4)	0.034 (4)	−0.001 (3)	0.008 (3)	−0.005 (3)
C35	0.040 (4)	0.042 (4)	0.042 (4)	0.002 (3)	0.022 (3)	−0.009 (3)
C36	0.031 (4)	0.039 (4)	0.038 (4)	−0.002 (3)	0.021 (3)	−0.004 (3)
C41	0.020 (3)	0.025 (3)	0.023 (3)	0.003 (2)	0.008 (3)	−0.002 (2)
C42	0.033 (4)	0.021 (3)	0.037 (4)	−0.001 (3)	0.018 (3)	−0.003 (3)
C43	0.033 (4)	0.039 (4)	0.047 (4)	−0.004 (3)	0.017 (3)	−0.007 (3)
C44	0.025 (4)	0.047 (5)	0.041 (4)	0.006 (3)	0.006 (3)	−0.006 (3)
C45	0.038 (4)	0.039 (4)	0.032 (4)	0.011 (3)	0.009 (3)	0.008 (3)
C46	0.025 (3)	0.031 (4)	0.030 (3)	0.004 (3)	0.011 (3)	0.011 (3)
C51	0.032 (4)	0.064 (5)	0.041 (4)	−0.009 (4)	0.007 (3)	−0.001 (4)

### Geometric parameters (Å, °)

**Table:**

I1—Rh1	2.6920 (6)	C15—C16	1.386 (9)
I2—Rh1	2.6743 (6)	C15—H15	0.9500
Rh1—C4	1.996 (6)	C16—H16	0.9500
Rh1—P2	2.3185 (15)	C21—C26	1.391 (8)
Rh1—P1	2.3248 (15)	C21—C22	1.396 (8)
Cl1—C51	1.760 (7)	C22—C23	1.365 (9)
Cl2—C51	1.761 (7)	C22—H22	0.9500
P1—C21	1.826 (6)	C23—C24	1.383 (9)
P1—C1	1.827 (5)	C23—H23	0.9500
P1—C11	1.830 (6)	C24—C25	1.364 (9)
P2—C41	1.821 (6)	C24—H24	0.9500
P2—C31	1.821 (6)	C25—C26	1.378 (9)
P2—C3	1.832 (6)	C25—H25	0.9500
F1—C4	1.353 (7)	C26—H26	0.9500
F2—C4	1.428 (7)	C31—C32	1.378 (8)
F3—C5	1.362 (8)	C31—C36	1.394 (8)
F4—C5	1.351 (7)	C32—C33	1.380 (9)
F5—C6	1.317 (8)	C32—H32	0.9500
F6—C6	1.345 (8)	C33—C34	1.379 (9)
F7—C6	1.367 (9)	C33—H33	0.9500
C1—C2	1.518 (8)	C34—C35	1.384 (9)
C1—H1A	0.9900	C34—H34	0.9500
C1—H1B	0.9900	C35—C36	1.376 (9)
C2—C3	1.517 (8)	C35—H35	0.9500
C2—H2A	0.9900	C36—H36	0.9500
C2—H2B	0.9900	C41—C46	1.392 (8)
C3—H3A	0.9900	C41—C42	1.399 (8)
C3—H3B	0.9900	C42—C43	1.387 (9)
C4—C5	1.562 (9)	C42—H42	0.9500
C5—C6	1.522 (10)	C43—C44	1.373 (10)
C11—C12	1.390 (8)	C43—H43	0.9500
C11—C16	1.390 (8)	C44—C45	1.375 (9)
C12—C13	1.397 (8)	C44—H44	0.9500
C12—H12	0.9500	C45—C46	1.385 (8)
C13—C14	1.368 (9)	C45—H45	0.9500
C13—H13	0.9500	C46—H46	0.9500
C14—C15	1.391 (9)	C51—H51A	0.9900
C14—H14	0.9500	C51—H51B	0.9900
			
C4—Rh1—P2	89.82 (18)	C13—C14—H14	119.9
C4—Rh1—P1	96.10 (19)	C15—C14—H14	119.9
P2—Rh1—P1	92.02 (5)	C16—C15—C14	119.5 (6)
C4—Rh1—I2	99.44 (18)	C16—C15—H15	120.2
P2—Rh1—I2	88.04 (4)	C14—C15—H15	120.2
P1—Rh1—I2	164.46 (4)	C15—C16—C11	120.8 (6)
C4—Rh1—I1	103.92 (17)	C15—C16—H16	119.6
P2—Rh1—I1	166.17 (4)	C11—C16—H16	119.6
P1—Rh1—I1	88.17 (4)	C26—C21—C22	117.1 (6)
I2—Rh1—I1	88.105 (17)	C26—C21—P1	121.7 (5)
C21—P1—C1	104.1 (3)	C22—C21—P1	121.1 (4)
C21—P1—C11	105.5 (3)	C23—C22—C21	121.5 (6)
C1—P1—C11	101.2 (3)	C23—C22—H22	119.2
C21—P1—Rh1	107.23 (19)	C21—C22—H22	119.2
C1—P1—Rh1	116.1 (2)	C22—C23—C24	120.0 (6)
C11—P1—Rh1	121.06 (19)	C22—C23—H23	120.0
C41—P2—C31	102.8 (3)	C24—C23—H23	120.0
C41—P2—C3	102.2 (3)	C25—C24—C23	119.7 (6)
C31—P2—C3	104.1 (3)	C25—C24—H24	120.1
C41—P2—Rh1	121.14 (19)	C23—C24—H24	120.1
C31—P2—Rh1	109.46 (19)	C24—C25—C26	120.3 (6)
C3—P2—Rh1	115.3 (2)	C24—C25—H25	119.8
C2—C1—P1	115.6 (4)	C26—C25—H25	119.8
C2—C1—H1A	108.4	C25—C26—C21	121.2 (6)
P1—C1—H1A	108.4	C25—C26—H26	119.4
C2—C1—H1B	108.4	C21—C26—H26	119.4
P1—C1—H1B	108.4	C32—C31—C36	118.2 (6)
H1A—C1—H1B	107.5	C32—C31—P2	122.0 (5)
C3—C2—C1	113.8 (5)	C36—C31—P2	119.8 (5)
C3—C2—H2A	108.8	C31—C32—C33	121.3 (6)
C1—C2—H2A	108.8	C31—C32—H32	119.3
C3—C2—H2B	108.8	C33—C32—H32	119.3
C1—C2—H2B	108.8	C34—C33—C32	119.9 (6)
H2A—C2—H2B	107.7	C34—C33—H33	120.0
C2—C3—P2	114.3 (4)	C32—C33—H33	120.0
C2—C3—H3A	108.7	C33—C34—C35	119.6 (6)
P2—C3—H3A	108.7	C33—C34—H34	120.2
C2—C3—H3B	108.7	C35—C34—H34	120.2
P2—C3—H3B	108.7	C36—C35—C34	120.1 (6)
H3A—C3—H3B	107.6	C36—C35—H35	120.0
F1—C4—F2	103.1 (4)	C34—C35—H35	120.0
F1—C4—C5	106.6 (5)	C35—C36—C31	120.8 (6)
F2—C4—C5	99.3 (5)	C35—C36—H36	119.6
F1—C4—Rh1	114.9 (4)	C31—C36—H36	119.6
F2—C4—Rh1	112.0 (4)	C46—C41—C42	118.7 (6)
C5—C4—Rh1	118.8 (4)	C46—C41—P2	119.5 (4)
F4—C5—F3	110.5 (5)	C42—C41—P2	121.7 (5)
F4—C5—C6	106.2 (6)	C43—C42—C41	120.6 (6)
F3—C5—C6	103.9 (6)	C43—C42—H42	119.7
F4—C5—C4	110.9 (5)	C41—C42—H42	119.7
F3—C5—C4	108.4 (5)	C44—C43—C42	120.1 (6)
C6—C5—C4	116.7 (6)	C44—C43—H43	120.0
F5—C6—F6	110.1 (6)	C42—C43—H43	120.0
F5—C6—F7	106.6 (6)	C43—C44—C45	119.7 (6)
F6—C6—F7	102.9 (6)	C43—C44—H44	120.1
F5—C6—C5	113.8 (6)	C45—C44—H44	120.1
F6—C6—C5	110.0 (6)	C44—C45—C46	121.2 (6)
F7—C6—C5	112.8 (6)	C44—C45—H45	119.4
C12—C11—C16	119.2 (6)	C46—C45—H45	119.4
C12—C11—P1	120.6 (5)	C45—C46—C41	119.7 (6)
C16—C11—P1	120.2 (5)	C45—C46—H46	120.2
C11—C12—C13	119.8 (6)	C41—C46—H46	120.2
C11—C12—H12	120.1	Cl1—C51—Cl2	112.3 (4)
C13—C12—H12	120.1	Cl1—C51—H51A	109.2
C14—C13—C12	120.5 (6)	Cl2—C51—H51A	109.2
C14—C13—H13	119.7	Cl1—C51—H51B	109.2
C12—C13—H13	119.7	Cl2—C51—H51B	109.2
C13—C14—C15	120.2 (6)	H51A—C51—H51B	107.9
